# Caviomorph rodents from the Pampean region (Argentina) in the historical Santiago Roth Collection in Switzerland

**DOI:** 10.1186/s13358-023-00272-8

**Published:** 2023-05-17

**Authors:** Leonardo Kerber

**Affiliations:** 1 Centro de Apoio à Pesquisa Paleontológica da Quarta Colônia, Universidade Federal de Santa Maria (CAPPA/UFSM), Rua Maximiliano Vizzotto, 598, São João do Polêsine, RS 97230-000 Brazil; 2 Programa de Pós-Graduação em Biodiversidade Animal, Universidade Federal de Santa Maria, Av. Roraima, 1000, Santa Maria, RS 97105-900 Brazil

**Keywords:** Chinchillidae, Caviidae, Echimyidae, Quaternary, Historical collections, Chinchillidae, Caviidae, Echimyidae, Cuaternario, Colecciones históricas

## Abstract

Here I reviewed the Pleistocene caviomorphs collected by Santiago Roth (collection from Catalog No. 5) and housed at the paleontological collection of the Paläontologisches Institut und Museum, Universität Zürich, Zurich (Switzerland). The fossils were found in Pleistocene strata from Buenos Aires and Santa Fé provinces (Argentina) during the late nineteenth century. The material includes craniomandibular remains assigned to *Lagostomus maximus* (Chinchilloidea: Chinchillidae), craniomandibular and postcranial (thoracic and sacral vertebra, left scapula, left femur, and right tibia) bones identified as *Dolichotis* sp. (Cavioidea: Caviidae), and a fragmented hemimandible and isolated tooth of *Myocastor* sp. (Octodontoidea: Echimyidae). Other rodent specimens from this collection (*Ctenomys* sp. and *Cavia* sp.) are possibly sub-recent materials.

## Resumen

En este trabajo se estudian los caviomorfos del Pleistoceno colectados por Santiago Roth (colección del Catálogo Nro. 5) y conservados en la colección paleontológica del Paläontologisches Institut und Museum, Universität Zürich, Zurich (Switzerland). Los fósiles fueron recuperados de niveles del Pleistoceno de las provincias de Buenos Aires y Santa Fé (Argentina) hacia fines del siglo 19. Los materiales incluyen restos craniomandibulares asignados a *Lagostomus maximus* (Chinchilloidea: Chinchillidae), elementos craniomandibulares y postcraneanos (vértebras torácicas y sacras, escápula izquierda, femúr izquierdo y tibia derecha), identificados como *Dolichotis* sp. (Cavioidea: Caviidae), y un fragmento de una hemimandíbula y un diente aislado de *Myocastor* sp. (Octodontoidea: Echimyidae). Otros roedores de esta colección (*Ctenomys* sp. y *Cavia* sp.) posiblemente son materiales subrecientes.

## Introduction

The evolutionary history of the South American rodents—Caviomorpha—in deep time has been revealed through interpretations from the fossil record (Vucetich et al., 2015). These curious mammals have evolved on this continent probably since the Middle Eocene (Antoine et al., 2012; but see Campbell et al., 2021), and today they represent a significant part of the local endemic diversity of mammals, with almost 250 species (Patton et al., 2015). The high diversification of these rodents, associated with ecomorphological adaptations, promoted the colonization of most environments likely inhabited in this land mass (Patton et al., 2015; Upham & Patterson, 2015).

The diversity of extant and extinct crown Caviomorpha is included in four major clades: Erethizontoidea, Cavioidea, Chinchilloidea, and Octodontoidea (D’Elia et al., 2019; Fabre et al., 2015; Upham & Patterson, 2015). Fossils of caviomorphs indicate that these groups were already split during the Paleogene. At the same time, some enigmatic taxa are not included in such groups and probably represent extinct stem lineages (see Frailey & Campbell, 2004; Antoine et al., 2012; Bertrand et al., 2012; Vucetich et al., 2015; Boivin et al., 2016, 2017, 2018, 2019; Arnal et al., 2019, 2022). Neogene was an interval of time of great importance in the diversification of caviomorphs, evidenced by the disparity in body sizes that marked the evolution of several groups (Ferreira et al., 2020; Rinderknecht & Blanco, 2008; Sánchez-Villagra et al., 2003). Several groups became extinct in this continent during the Late Miocene and Pliocene, possibly associated with drastic environmental and biotic changes (Vucetich et al., 1999, 2015). In addition, during the Pliocene the fossil record is marked by the appearance of several fossils assigned to extant genera (e.g., Candela & Boinini, 2018; Madozzo-Jaén et al., 2021). Subsequently, during Pleistocene times, a significant part of the current caviomorph diversity was already established, at least at the generic level. However, some genera and species became extinct (some closely related to extant forms), and other taxa had a geographic distribution different from current biogeographic patterns (e.g., Tonni, 1981; Vucetich & Verzi, 1999, 2002; Vucetich et al., 1997, 1999, 2015; Ubilla & Rinderknecht, 2001, 2014, 2016; Ubilla et al., 2008; Kerber et al., 2011a, 2011b, 2014, 2016, 2020; Mayer et al., 2016; Kerber, 2017; Vezzozi & Kerber, 2017; Eduardo et al., 2018; Gomes et al., 2019).

Fossils from the Pampean region of Argentina (including rodents) have been fundamental for understanding the Pleistocene biota of South America. The Swiss/Argentinean paleontologist Santiago Roth (1850–1924) was one of the main contributors to the rapid increase of knowledge on these fossils during the late 1800s and early 1900s (Machon, 1925; Weigelt, 1951; Giacchino & Gurovich, 2001; Sánchez-Villagra et al., 2023). Here I reviewed the fossils of caviomorph rodents collected by Roth and housed at the Paläontologisches Institut und Museum, Universität Zürich, Zurich (Switzerland) (collection nº 5) (Roth, 1889).

## Material and methods

### Collection and provenance

The analyzed specimens were collected from Pleistocene strata in the Buenos Aires and Santa Fé provinces (Argentina) (see below). They are housed at the paleontological collection of the Paläontologisches Institut und Museum, Universität Zürich, Zurich (Switzerland). The specimens, originally numbered in Roth (1889), have recently been assigned new collection numbers with the acronym PIMUZ A/V. The stratigraphic provenance of the specimens is imprecise. Roth (1889) identified the strata as “*Pampéen inférieur, Moyen, and Supérieur*”, which would be rougly equivalent to the Early, Middle, and Upper Pleistocene (Cione & Tonni, 1999). See Voglino et al. (2023) for further details.

### Nomenclature and measurements

For cranial anatomy, I employed the set of terms compiled by Kerber et al. (2019a) from several sources (e.g., Moore, 1981; Novacek, 1985, Wahlert, 1985; Wilson & Sánchez-Villagra, 2009; Nasif & Abdala, 2015). Description of the auditory region follows the recent study of the caviomorph ear by Arnaudo et al. (2020), who compiled anatomical terms from different contributions (i.e., Wible et al., 2005, Wible, 2010), and Wible and Shelley (2020). For the dentary and lower cheek teeth of the caviids, I followed Pérez (2010). Postcranial anatomy was based on Candela and Picasso (2008) and references therein. Regarding terms of direction, I followed the recommendations of Nomina Anatomica Veterinaria (NAV, 2017): rostral, caudal, dorsal, and ventral for head structures. Measurements were taken with a digital caliper, following Kerber et al., (2019b) for the chinchillid cheek teeth, and Candela and Picasso (2008) for postcranial bones.

*Institutional abbreviations*. **MACN-A**, paleontological collection (Ameghino Collection) of the Museo Argentino de Ciencias Naturales Bernardino Rivadavia, Buenos Aires, Argentina; **PIMUZ/AV**, Paläontologisches Institut und Museum, Universität Zürich, Zurich, Switzerland.

## Systematic paleontology

Mammalia Linnaeus, 1758.

Rodentia Bowdich, 1821.

Hystricognathi Tullberg, 1899.

Chinchilloidea Kraglievich, 1940.

Chinchillidae Bennet, 1833.

*Lagostomus* Brookes, 1828.

*Lagostomus maximus* (Desmarest, 1817).

*Referred specimens*. PIMUZ A/V 4147, cranium and mandible, preserving the upper and lower cheek teeth series (Catalog No. 5, specimen 253); PIMUZ A/V 4235a, right dentary with cheek teeth (Catalog No. 5, specimen 254); PIMUZ A/V 4235b, right dentary with cheek teeth (Catalog No. 5, specimen 255); PIMUZ A/V 4202, fragmented left hemimandible with cheek teeth (Catalog No. 5, specimen 255).

*Provenance*. PIMUZ A/V 4147, Arroyo Pergamino, Buenos Aires, Argentina (*Pampéen Supérieur*, Roth, 1889); PIMUZ A/V 4235 a and b, Barranca Villa Constitución, Santa Fé, Argentina (*Pampéen Moyen*, Roth, 1889); PIMUZ A/V 4202, Barranca Villa Constitución, Santa Fé, Buenos Aires, Argentina (*Pampéen Moyen*, Roth, 1889).

**Fig. 1:**
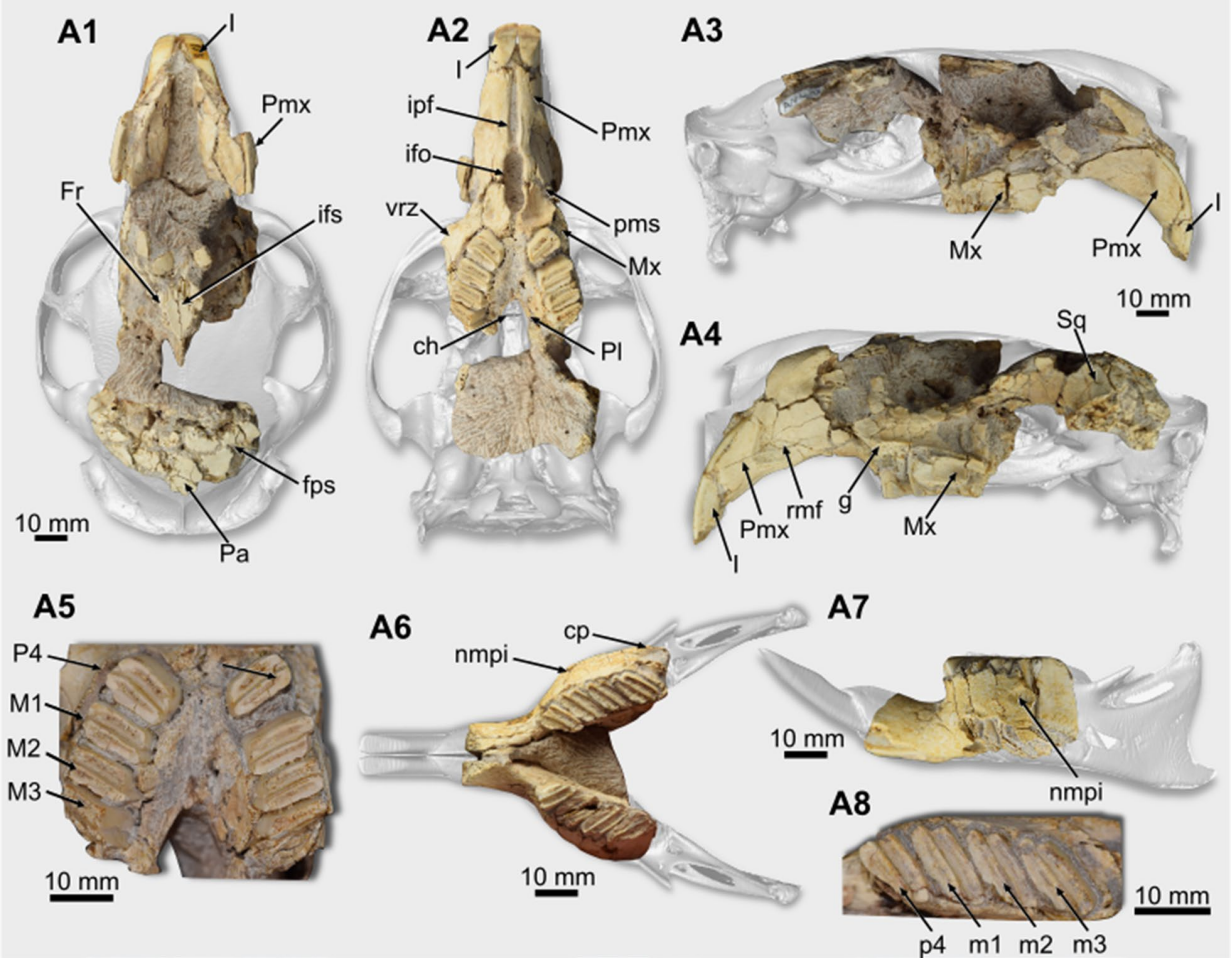
Cranial and mandibular remains of *Lagostomus maximus* (**A** PIMUZ A/V 4147). **A1**–**A4** cranium in dorsal (**A1**), ventral (**A2**), right lateral (**A3**), and left lateral (**A4**) views. **A5** upper cheek teeth series, in occlusal view. **A6**–**A7** mandibular remains, in occlusal and lateral views. **A8** detail of the right cheek teeth series. Abbreviations: ch, choana (or mesopterygoid fossa); cp, coronoid process; fps, frontoparietal suture; Fr, frontal; g, groove; I, upper incisor; ifo, incisive foramen; ifs, interfrontal suture; ipf, interpremaxillary foramen; nmpi, notch for the insertion of the muscle *masseter medialis pars infraorbitalis*; Mx, maxilla; M1–M3, first to third upper molars; m1-m3, first to third lower molars; Pa, parietal; Pl, palatine; pms, premaxillo-maxillary suture; Pmx, premaxilla; P4, upper fourth premolar; p4, lower fourth premolar; Sq, squamosal; rfm, rostral masseteric fossa; vrz, ventral zygomatic root

*General description.* *Cranium*. The dorsal portion of the cranium is quite damaged (Fig. 1A1). The rostrodorsal region of both premaxillae is not preserved, and the nasals and zygomatic arches are missing (Fig. 1A1–A4). There are frontal fragments in which the suture between both bones (interfrontal) and the frontoparietal suture are discernible (Fig. 1A1). This latter is interdigitated and laterally oriented. Parietal bones are cracked, and it is not possible to describe them. The specimen conserves a portion of the right squamosal, forming the caudodorsal limit of the orbit (Fig. 1A4).

In the rostral region of the cranium, the diastema is long (Table 1), and its ventral surface is concave. However, the concavity is not accentuated (Fig. 1A2–A4). The premaxilla is one of the best preserved cranial bones. On its lateral face is a well-marked rostral masseteric fossa, which is deeper in its rostral region (Fig. 1A4). Dorsally, this fossa is delimited by a pronounced rostral masseteric crest. This crest is slightly curved and is rostroventrally oriented. Ventrally, in the rostral region of the diastema, between both premaxillae, there is a lenticular, rostrocaudally oriented, interpremaxillary foramen (Fig. 1A2). Caudally to this foramen is a wide incisive foramen. This foramen is oval in outline but laterally compressed. The premaxillo-maxillary suture crosses the foramen at its middle portion (Fig. 1A2). Lateral to the caudalmost region of the foramen, there is a shallow fossa on the maxilla’s ventral surface.

**Table 1:** Measurements (in mm) of the fossils of *Lagostomus maximus*

Measurements	PIMUZ A/V 4147	PIMUZ A/V 4147	PIMUZ A/V 4235a	PIMUZ A/V 4235b	PIMUZ A/V 4202
Cranium and upper cheek teeth		–	–	–	–
Diastema length	41.26	–	–	–	–
Height of the rostral masseteric fossa	28.0	–	–	–	–
Interpremaxillary foramen length	20.7	–	–	–	–
Incisive foramen length	19.8	–	–	–	–
Rostrocaudal length of the ventral zygomatic root	11.28	–	–	–	–
Width between both p4s	6.4	–	–	–	–
Upper Incisor width	6.1	–	–	–	–
MDL of the P4	5.36	–	–	–	–
LLW of the P4	9.44	–	–	–	–
MDL of the M1	6.08	–	–	–	–
LLW of the M1	10.02	–	–	–	–
MDL of the M2	6.63	–	–	–	–
LLW of the M2	9.55	–	–	–	–
LLW of the M3	8.6	–	–	–	–
Lower teeth					
p4-m3 series length	–	27.7	23.06	23.59	25.28
MDL of the p4	–	5.58	3.76	4.09	–
LLW of the p4	–	9.55	6.68	6.54	–
MDL of the m1	–	6.18	4.50	–	5.40
LLW of the m1	–	10.5	7.58	6.83	8.87
MDL of the m2	–	6.54	4.68	4.70	5.23
LLW of the m2	–	10.8	7.24	8.25	7.83
MDL of the m3	–	6.17	4.80	4.99	–
LLW of the m3	–	9.79	7.77	8.61	–

*MDL* mesiodistal length, *LLW* labiolingual width

On the lateral face of the maxilla, the ventral zygomatic root is at the level of the P4-M1. It is broken at its base (Fig. 1A2). Dorsally to this structure, there is a groove delimited laterally by a thin lamina (Fig. 1A4).

The palatal region, composed of the palatal processes of the maxilla and palatine, is concave (Fig. 1A2). This region preserves both dental series (see below), which are caudally divergent, forming an angle of ~ 26º concerning the sagittal plane. Rostrally, lateral to the suture between both maxillae, there is a rostrocaudally oriented palatal sulcus (Fig. 1A2). On the caudal region of the palate, the specimen preserves part of the palatines (Fig. 1A2), and the maxillo-palatine suture can be partially observed.

**Fig. 2:**
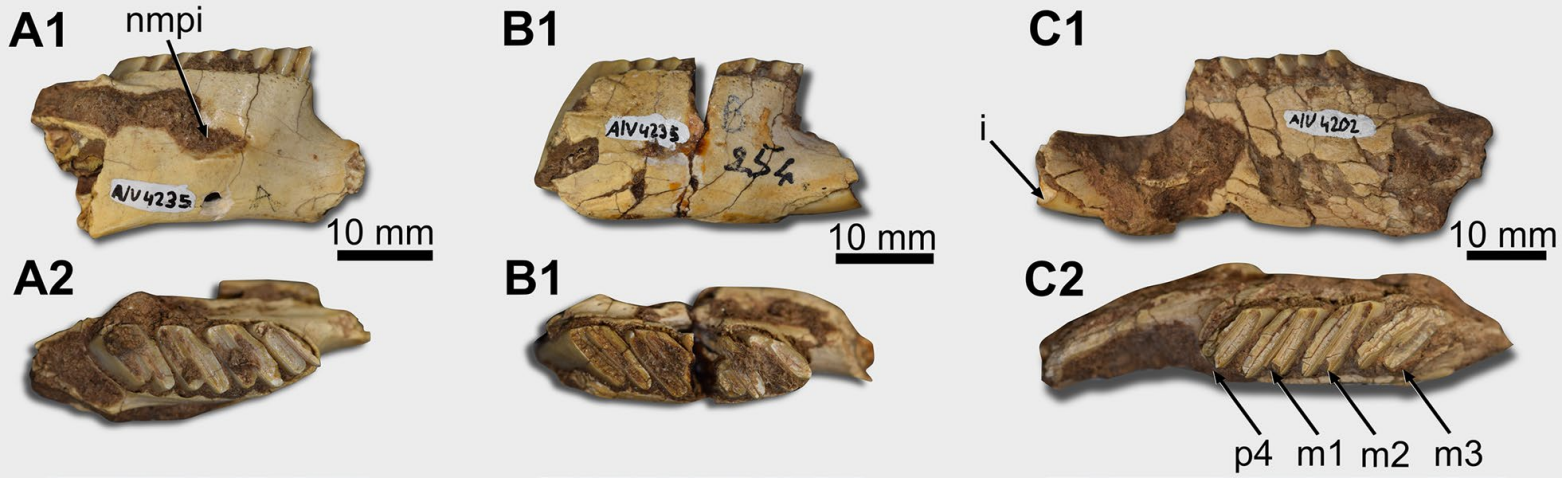
Mandibular remains and lower teeth of *Lagostomus maximus* (**A** PIMUZ A/V 4235a, **B** PIMUZ A/V 4235b, **C** PIMUZ A/V 4202). **A1**–**A2** right hemimandible, in lateral and occlusal views. **B1**–**B2** right hemimandible, in lateral and occlusal. **C1**–**C2** left hemimandible, in lateral and occlusal views. Abbreviations: I, lower incisor; nmpi, notch for the insertion of the muscle *masseter medialis pars infraorbitalis*; m1-m3, first to third lower molars; p4, fourth lower premolar

*Mandible. *PIMUZ A/V 4147 and PIMUZ A/V 4235 a and b preserve the rostral portion of the dentary bodies, missing the region caudal to the m3 (Fig. 1A6–A7, 2A1–B2). At the same time, PIMUZ A/V 4202 is more complete, but its surface is damaged (Fig. 2C1–C2). On the right dentary of PIMUZ A/V 4147, which is better preserved, the notch for the insertion of the muscle *masseter medialis pars infraorbitalis* is placed at the level of the m1-m2 (Fig. 1A6). The base of the coronoid process is lateral to the distal region of the m3 (Fig. 1A6). Ventrally to the coronoid process is the rostralmost region of the masseteric fossa and a portion of the horizontal crest delimiting it laterally.

*Teeth*. In PIMUZ A/V 4147, the upper incisors are complete (Fig. 1A1–A4). They are proodont and have a subtriangular section. The enamel covers the labial surface, which is flat and marked by fine longitudinal striations. The distal corner of the incisor is curved, while the mesial forms a right border. The lower ones are broken, remaining only their base.

The cheek teeth (Table 1) of the analyzed specimens are euhypsodont (sensu Mones, 1982). The upper series of PIMUZ A/V 4147 preserves the P4–M2 series (Fig. 1A5). The P4, M1, and M2 are morphologically similar to each other. They comprise two oblique laminae separated by a very thin lingual flexus. The angle of the tooth laminae concerning the sagittal plane is 48.8º. The mesial enamel layer that surrounds each lamina is thicker than the distal. Only the first lamina is preserved in both teeth from the M3.

PIMUZ A/V 4147 preserves the p4–m3 series (Table 1) (Fig. 1A6–A8), better preserved on the right side. PIMUZ A/V 4235a preserves the p4–m3 series (Table 1) (Fig. 2A), except for the broken first lophid of the p4. In PIMUZ A/V 4235b, the m1 is broken ( Fig. 2B). The lower cheek teeth are bilophododont and a labial flexid separates the laminae. The distal enamel layer is thicker than the mesial. The angle of the laminae (following Rasia & Candela, 2013) is 25º. PIMUZ A/V 4202 conserves the p4–m3 series (Fig. 2C). However, only the second lophid of the left p4 and the m1 and m2 have the occlusal surfaces preserved.

**Fig. 3:**
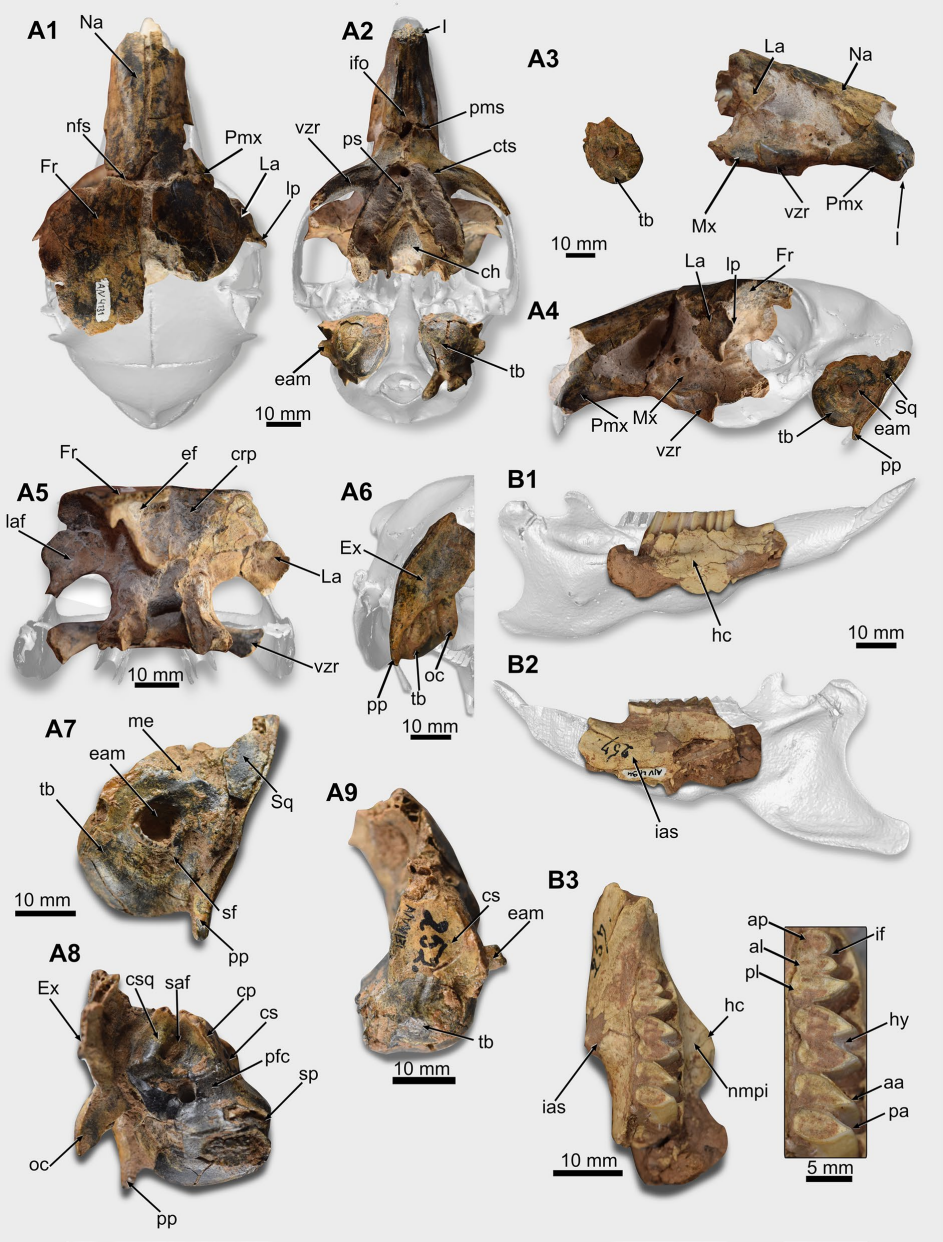
Cranial and mandibular remains of *Dolichotis* sp. (**A** PIMUZ/AV 4131;** B** PIMUZ/AV 4194). **A1**–**A6** rostral region of the cranium, in dorsal (**A1**), ventral (**A2**), right lateral (**A3**), left lateral (**A4**), and caudal (**A5**) views. **A6**, occipital region of the cranium, in caudal view. **A7**–**A9** right ear region, in lateral (**A7**), medial (**A8**), and rostral (**A9**) views. **B1**–**B3** right dentary, in lateral (**B1**), medial (**B2**), and occlusal (**B3**) views (with the cheek teeth series in detail). Abbreviations: aa, anterior apex; al, anterior lobe; ap, anterior projection; ch, choana; cp, crista petrosa; crp, cribriform plate; cs, cerebellar surface; csq, crista squamosa; cts, cheek teeth alveolar series (P4-M3); eam, external acoustic meatus; ef, ethmoidal fossa; Ex, exoccipital; Fr, frontal; I, upper incisor; ias, incisive alveolar sheath; if, interprismatic furrow; ifo, incisive foramen; hc, horizontal crest; hy, hypoflexid; La, lacrimal; laf, lacrimal foramen; lp, lacrimal process; Na, nasal; nfs, nasofrontal suture; me, mastoid exposure; nmpi, notch for the insertion of the muscle *masseter medialis pars infraorbitalis*; Mx, maxilla; saf, subarcuate fossa; sf, stylomastoid foramen; sp, styliform process; oc, occipital condyle; pa, posterior apex; pfc, prefacial commissure; pl, posterior lobe; pms, premaxillo-maxillary suture; Pmx, premaxilla; pp, paroccipital process; ps, palatal sulcus; Sq, squamosal; tb, tympanic bulla (ectotympanic); vzr, ventral zygomatic root

*Remarks*. *Lagostomus maximus*, the vizcacha, is a chichillid rodent that inhabits open areas in central, north, and northwest Argentina, western Paraguay, and southern Bolivia (Jackson et al., 1996; Llanos & Crespo, 1952; Spotorno & Patton, 2015). This taxon is also present in the Pleistocene fossil record of Argentina, including some places that they do not inhabit today, such as Uruguay and southern Brazil (*e.g*., Tonni & Fidalgo, 1982; Prado et al., 1987; Tonni et al., 1988; Gómez et al., 1999; Sarrat, 2009; Cruz et al., 2009; Kerber et al., 2011a; Ubilla & Rinderknecht, 2016). According to the comprehensive review of the Pleistocene fossil record of *Lagostomus* by Ubilla and Rinderknecht (2016), the species *L. maximus* is the only confidently valid taxon (however, according to Rasia, 2021, *L. incisus* is recorded in the Raigón Formation, at levels that could be of Pleistocene age). The material reported here is assigned to the *L. maximus* based on the presence of bilaminar and euhypsodont cheek teeth, and angle of the lamina of the upper cheek teeth concerning the sagittal plane compatible with the species (Rasia & Candela, 2013, 2017a, 2007b; Ubilla & Rinderknecht, 2016; Rasia et al., 2020; Rasia, 2021).

Cavioidea (Fischer, 1817) *sensu* Kraglievich 1930

Caviidae (Fischer, 1817) *sensu* Waterhouse 1839

Dolichotinae Pocock, 1922

*Dolichotis* Desmarest, 1820

*Dolichotis* sp.

*Referred specimens.* PIMUZ/AV 4131, cranium without teeth and postcranial remains (thoracic and sacral vertebra, left scapula, left femur, and right tibia) (Catalog No. 5, specimen 252); PIMUZ/AV 4194, right dentary with p4–m2 (Catalog No. 5, specimen 257).

*Provenance*. PIMUZ/AV 4131, San Nicolas, Buenos Aires, Argentina (*Pampéen Moyen*, Roth, 1889); PIMUZ/AV 4194, Arroyo Pergamino, Buenos Aires, Argentina (*Pampéen Supérieur*, Roth, 1889).

*General description*. *Cranium*. The rostral half of the cranium of PIMUZ/AV 4131 is preserved (Fig. 3A1–A9; Table 2). The region caudal to the palate is missing, but isolated left and right ears, and a portion of the occiput are preserved.

**Table 2:** Measurements (in mm) of the fossils of *Dolichotis* sp. (femur dimensions taken according to Candela & Picasso, 2008)

Measurements	PIMUZ/AV 4131	PIMUZ/AV 4194
Cranium		
Diastema length	35.87	–
P4-M3 series length	28.27	–
Width between both p4s	1.7	–
Incisor width	4.06	–
Nasal length	41.76	–
Nasal width	4.83	–
Interpremaxillary foramen length	27.15	–
Rostrocaudal length of the ventral zygomatic root	11.28	–
Maximum width of the choana	40.42	–
Length of the tympanic bulla	18.8	–
Diameter of the external acoustic meatus	7.77	–
Length of the subarcuate fossa	8.58	–
Width of the subarcuate fossa	6.25	–
Length of the paroccipital process	16.75	–
Lower teeth		
MDL of the p4	–	7.01
LLW of the p4	–	5.30
LLW of the anterior projection of the p4	–	2.96
MDL anterior lobe of the p4	–	3.89
LLW anterior lobe of the p4	–	5.28
MDL of the m1	–	6.64
LLW of the m1	–	5.83
MDL of the m2	–	6.69
LLW of the m2	–	5.95
Scapula		
Length of the glenoid fossa	18.84	–
Width of the glenoid fossa	13.81	–
Femur	132	–
Femoral functional length	123.36	–
Transverse diameter of the mid-shaft of the femur	11.15	–
Femoral medial condyle width	10.45	–
Femoral lateral condyle width	10.70	–
Femoral head width	13.50	–
Femoral head length	13.53	–
Femoral distal depth	29.96	–
Femoral distal end width	24.70	–
Tibia		
Craniocaudal length of the proximal region of the tibia	21.55	–
Transversal width of the proximal region of the tibia	23.79	–

*MDL* mesiodistal length, *LLW* labiolingual width

PIMUZ/AV 4131 shows a narrow rostrum with a long diastema, larger than the upper dental series (Table 2) (Fig. 3A2–A4). The lateral face of the rostrum is damaged (Fig. 3A3–A4). On the right side of the rostrum, there is a fragment of the caudalmost region of the premaxilla, contacting the frontal (Fig. 3A1). The rostral part of the nasal is transversely curved, and its caudal end has a flat surface. Its extension is uniform in width except at its rostralmost point, in which it tapers to form the nasal process. The nasal suture is straight and rostrocaudally elongated (Fig. 3A1). On the ventral region of the rostrum, there is a long incisive foramen (possibly confluent with the interpremaxillary foramen). It is narrow and lenticular in shape. The premaxillo-maxillary suture crosses the foramen transversely at is caudal portion (Fig. 3A2).

On the lateral side of the maxilla, the base of the ventral zygomatic root is at the level of the P4–M1 (Fig. 3A2–A4). This root is laterocaudally oriented. On the dorsal face of the ventral zygomatic root, there is a groove limited laterally by a ridge.

The palatal region is triangular, and the dental series are divergent caudally (Fig. 3A2). The maxillo-palatine suture is visible parallel to the dental series. The palatine forms the caudal end of the palatal region and the rostral edge of the choana or mesopterygoid fossa. This one is V-shaped, and its rostralmost point is at the level of the M2 (Fig. 3A2).

The orbital region is wide. Medially, this region is formed by the frontal, ventrally by the maxilla, and the lacrimal forms its rostrolateral edge. The lacrimal is well preserved on both sides of the cranium (Fig. 3A3–A5). It displays a well-developed lacrimal process that is laterocaudally oriented. The lacrimal foramen is on the caudal face of this process (Fig. 3A5). The frontal is wide and flat and forms the roof of the orbital region. The suture between the frontals is artificially opened by the taphonomic processes. A triangular frontal projection penetrates between the nasal and premaxilla on its anteriormost region.

The ethmoidal fossae of PIMUZ/AV 4131 for the olfactory bulbs are visible in the caudal aspect of the preserved portion of the cranium (Fig. 3A5). They have the shape of an inverted right triangle. The dorsal and lateral limits of these fossae are formed by the frontal, and rostrally they are enclosed by the cribriform plate.

Both tympanic bullae (ectotympanic) of PIMUZ/AV 4131 are preserved (Fig. 3A2, A7–A9). The bulla is rounded and presents an external acoustic meatus surrounded by a bony tube laterodorsally oriented (Fig. 3A7). The stylomastoid foramen is placed caudoventrally to the external acoustic meatus but is filled by matrix. The rostralmost region of the styliform process of the ectotympanic is broken, and its tip is missing (Fig. 3A8). Caudally, the styliform process is continuous, with a curved crest that marks the limit between the mastoid exposure of the petrosal and the ectotympanic bulla.

Rostrally, the cerebellar surface of the petrosal is smooth, and there is a dorsoventrally tenuous ridge at the middle line of this surface (Fig. 3A9). In medial view, the subarcuate fossa is at the dorsal level of the mastoid exposure of the petrosal (Fig. 3A8). Most of it is filled by the sedimentary matrix. Caudally, it is delimited by the crista squamosa. Laterally, the fossa is delimited by a marked crista petrosa lateromedially oriented that extends from the caudalmost region of the subarcuate fossa, almost reaching the area of the styliform process of the ectotympanic (Fig. 3A8). This crest delimits the cerebellar surface medially. Rostrally to the subarcuate fossa and medially to the crista petrosa, the prefacial commissure is wide. The region of the internal acoustic meatus is damaged by an artificial hole (probably made for inserting a support) (Fig. 3A8).

The left bulla is associated with a fragment of the squamosal and with a part of the occiput preserving part of the exoccipital (Fig. 3A7). There is a thin paroccipital process that extends ventrally, surpassing the ventral most limit of the bulla. However, its tip is broken.

*Dentary*. PIMUZ/AV 4194 preserves the rostral region of the dentary (Fig. 3B1–B3; Table 2). The portion caudal to the m2 is missing. Laterally, this dentary shows a strong horizontal crest, and its rostralmost point is at the level between p4 and m1 (Fig. 3B1). Medially, the incisive alveolar sheath forms a protuberance on the surface of the dentary that extends to the posterior lobe of the m1 (Fig. 3B2).

*Teeth*. Only the base of the incisors are preserved in PIMUZ/AV 4131 (Fig. 3A2–A3). Their mesial surface is flat, but the distal corner is rounded. Cheek teeth are not preserved, but the alveolar outline indicates the presence of P4–M2 formed by two lobes and the M3 with two lobes plus a posterior projection (Fig. 3A2).

Concerning the lower teeth, PIMUZ/AV 4194 preserves the p4–m2 (Fig. 3B3). They are euhypsodont, constituted of two main labially connected lobes and lanceolate lingual tips. The p4 shows the two main lobes (anterior and posterior) plus an anterior projection. This anterior projection is lingually rounded and separated from the anterior lobe by the interprismatic furrow (Fig. 3B3). The anterior and posterior lobes are transversely oriented. The anterior lobe is linguolabially shorter than the posterior. The hypoflexid, which separates both lobes, almost crosses the occlusal surface of the tooth. The labial side of the posterior lobe is slightly rounded. The m1 and m2 are similar. They are composed of two lobes with lanceolate lingual tips (anterior and posterior apexes, sensu Pérez, 2010) connected by a thin isthmus. Their apexes are slightly distally oriented compared to the lingual ones of the p4, which are transverse. The hypoflexid almost crosses the occlusal surface. Hence, both lobes are connected only by a thin and short isthmus. On the labial face, the lobes have a rounded outline and are separated by a shallow furrow (Fig. 3B3).

**Fig. 4:**
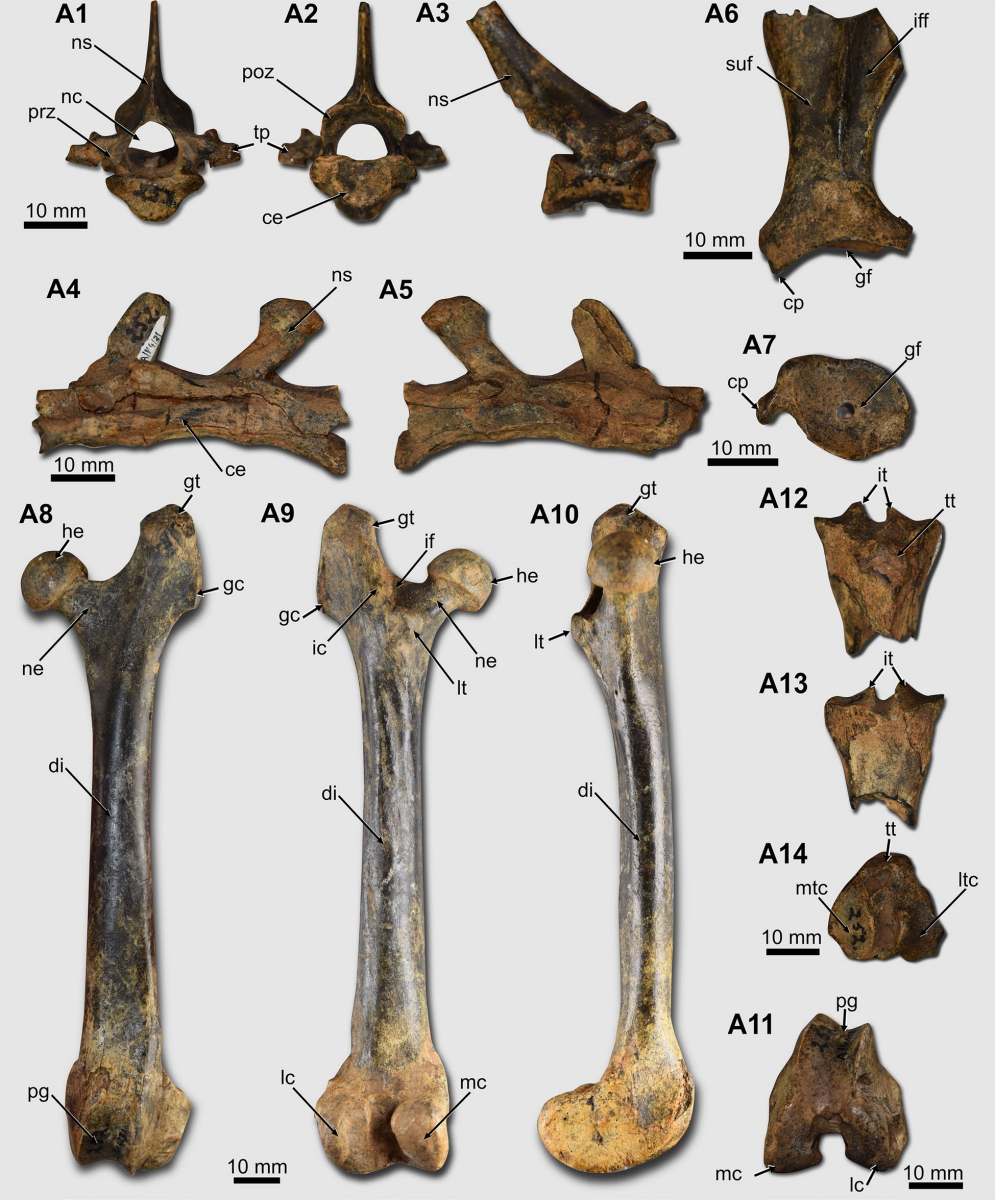
Postcranial remains of *Dolichotis* sp. (**A** PIMUZ/AV 4131). **A1**–**A3** thoracic vertebra, in cranial (**A1**), caudal (**A2**), and lateral (**A3**) views. **A4**–**A5** sacral vertebrae, in left lateral (**A4**) and right lateral (**A5**) views. **A6-A7** left scapula in lateral and distal views. **A8**–**A11** left femur, in cranial (**A8**), caudal (**A9**), medial (**A10**), and distal (**A11**) views. **A12-A14** right tibia, in cranial (**A12**), caudal (**A13**), and proximal (**A14**) views. Abbreviations: ce, centrum; cp, coracoid process; di, diaphysis; gc, gluteal crest; gf, glenoid fossa; gt, great trochanter; he, femoral head; ic, intertrochanteric crest; if, intertrochanteric fossa; iff, infraspinous fossa; it, intercondilar tuberosities; lc, lateral condyle; lt, lesser trochanter; ltc, lateral tibial condyle; mc, medial condyle; nc, neural canal; ne, neck; ns, neural spine; mtc, medial tibial condyle; pg, patellar groove; poz, postzygapophysis; prz, prezygapophysis; suf, supraspinous fossa; tp, transverse process; tt, tibial tubercle

*Postcranial skeleton*. PIMUZ/AV 4131 preserves a thoracic vertebra and a sequence of three sacral vertebrae (Fig. 4A1–A5). The thoracic vertebra is almost complete, missing only the tip of the neural spine (Fig. 4A1–A3).

The scapula of PIMUZ/AV 4131 does not preserve most of the scapular blade (Fig. 4A6–A7; Table 2). The coracoid process is short, and it is rostromedially oriented. A portion of the spine is preserved, observed in the external (or lateral) view. Caudally to the spine, PIMUZ/AV 4131 preserves part of the infraspinous fossa, which forms a depression on the scapular blade surface, distinct from the supraspinous fossa, in which there is no depression (Fig. 4A6). The glenoid fossa is shallow and has a pyriform outline, with a narrower region cranially (Fig. 4 A7).

The femur of PIMUZ/AV 4131 is complete (Fig. 4A8–A11; Table 2). In the proximal region of the femur, the femoral head is globose (Fig. 4 A8–A10). The fovea capitis is located on the mediocaudal region of the femoral head. The great trochanter is well-developed and surpasses the femoral head proximally. It is slightly laterally oriented. On its lateral side, there is a crest (gluteal crest) protruding laterally (Fig. 4A8–A9). The trochanteric fossa is deep. The lesser trochanter forms a rounded tuberosity that is mediocaudally oriented but is not visible in cranial view (Fig. 4A8). The intertrochanteric crest limits the trochanteric fossa caudally and is connected to the lesser trochanter (Fig. 4A9). No third trochanter is present. The diaphysis is long, gracile, and shows a slight variation in diameter in its extension (Fig. 4A8–A10). It is predominantly straight but has a slight craniocaudal curvature (Fig. 4A10). In the distal region, the patellar groove is slightly medially tilted (Fig. 4A8). Both lateral and medial condyles have approximately the same size. The craniocaudal diameter of the distal region of the femur is larger than the transverse diameter (Fig. 4A11).

The tibia only preserves its proximalmost region (Fig. 4A12–A14; Table 2). The lateral tibial condyle is broken on its lateral face. Both are transversely concave, but the lateral is more accentuated than the medial tibial condyle. The lateral condyle's tibial spine or intercondylar tuberosity projects more proximally than the medial one (Fig. 4A12–A13). In its cranial face, there is a preserved portion of the tibial tuberosity (Fig. 4A12).

*Remarks*. The genus *Dolichotis* encompasses two extant species: *Dolichotis patagonum*, from austral Argentina (Patagonia), and *Dolichotis salinicola*, from the Chaco ecoregion of Argentina, Paraguay, and Bolivia (Cabrera, 1961; Campos et al., 2001; Eisenberg & Redford, 1989; Madozzo-Jaén, 2019). The inclusion of both species in *Dolichotis* has been discussed over the years and recently confirmed by the comprehensive review by Madozzo-Jaén et al. (2021), who employed cladistic analysis, including morphological data of extant and extinct data and molecular data to analyze the phylogenetic relationships of dolichotines. Other four Quaternary species (*D. platycephala*, *D. intermedia*, *D. major*, and *D. minor*) were recognized by Ameghino (1889) from central Argentina. However, according to Madozzo-Jaén et al. (2021), these taxa were not studied after their original descriptions or other materials were not reported. Other Pleistocene records of *Dolichotis* are scarce and mainly represented by specimens not confidently identified at specific level. Most of these records of *Dolichotis* are from deposits in Argentina (Sarrat, 2009; Scillato-Yané et al., 1998; Tonni, 1981), but similar to the case of *Lagostomus* (see above), fossils out of the current distribution area are known from Uruguay and southern Brazil (Kerber et al., 2011a; Rodrigues & Ferigolo, 2004; Ubilla et al., 2004, 2009). Considering these facts, a taxonomic review of the Quaternary fossil record of *Dolichotis* is necessary to test if there are reliable extinct Quaternary taxa, or alternatively, if they are within the variation of the extant taxa, as in the case of *Lagostomus* extinct species (Ubilla & Rinderknecht, 2016).

The material described here shares morphological traits (e.g., expanded frontals, upper diastema longer than the cheek teeth series, choana reaching the M2, external acoustic meatus dorsolaterally oriented) with the species of the genus *Dolichotis* (Dunnum, 2015; Madozzo-Jaén, 2019; Madozzo-Jaén et al., 2021; Quintana, 1998; Ubilla & Rinderknecht, 2003), the largest non-hydrochoerine caviid. The length of the upper cheek teeth series is similar to the dimension of the specimen MACN-A 556 (*Dolichotis platycephala*) reported by Madozzo-Jaén et al. (2021), which is slightly larger than the average length in *D. patagonum* and smaller than the Pliocene *D. chapalmalense*. *Dolichotis salinicola* is the smallest species. The dimensions of the lower teeth of PIMUZ/AV 4194 are comparable with the data presented by Kerber et al. (2011a) and Madozzo-Jaén et al. (2021). They are compatible with *D. patagonum* (which has larger dimensions than *D. salinicola)*, and with the extinct *D. improla* and *D. intermedia* from central Argentina. The extinct Pliocene species *D. chapalmalense* has cheek teeth mesiodistally longer than our material and other specimens. Only one measure of *D. major* is comparable with our sample (length of the m2), being slightly larger. As the analyzed sample does not preserve the upper cheek teeth series (where important traits are present) and a taxonomic review of the extinct Quaternary species is needed, the specimens are not identified at specific level.

Octodontoidea Waterhouse, 1839

Echimyidae Gray, 1825

*Myocastor* Kerr, 1792

*Myocastor* sp.

*Referred specimens.* PIMUZ A/V 4204a, left dentary with fragmented m1 and m2 (Catalog No. 5, specimen 256); PIMUZ A/V 4204b, right m3. The isolated tooth may not be of the same individual as the dentary because there are differences in the preservation and ontogenetic differences.

*Provenance.* PIMUZ A/V 4204a and b, San Pedro, Buenos Aires Province, Argentina (*Pampéen Supérieur*, Roth, 1889).

**Fig. 5:**
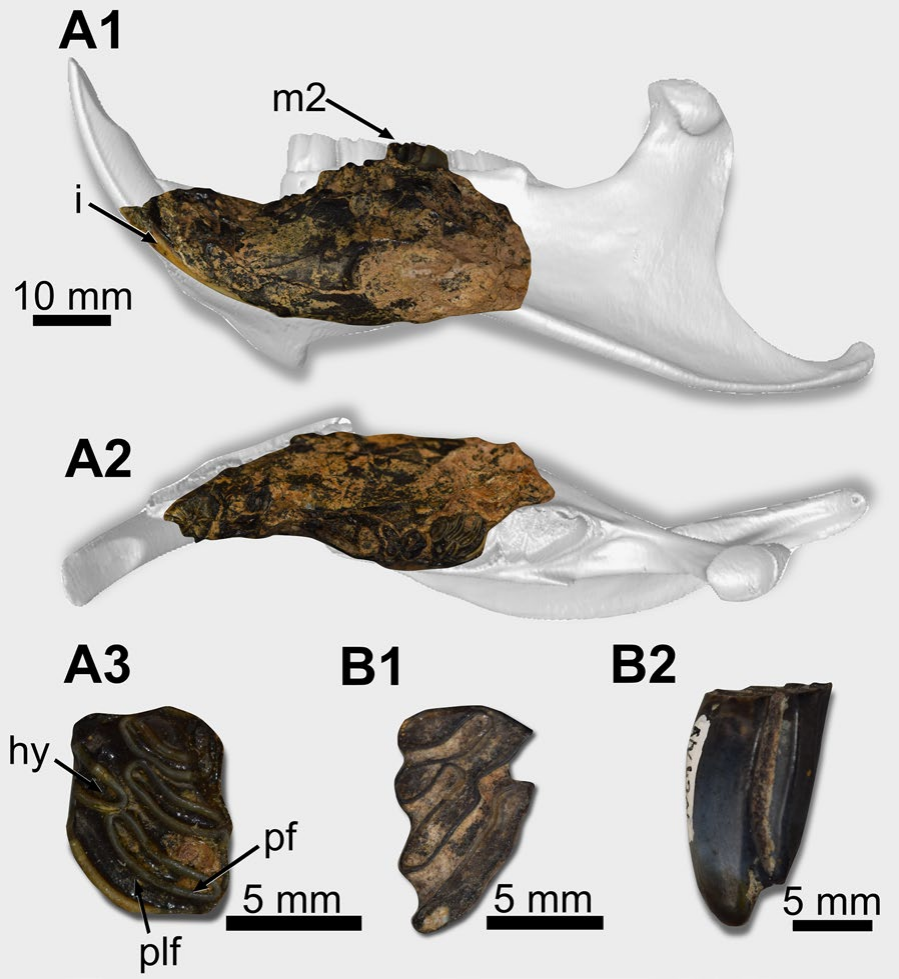
Mandibular and lower teeth remains of *Myocastor* sp. (**A** PIMUZ A/V 4204a, **B** PIMUZ A/V 4204b). **A1**–**A2** left hemimandible, in lateral (**A1**) and occlusal (**A2**) views. **A3** left m2, in occlusal view. **B1**–**B2** right m3, in occlusal (**B1**) and labial (**B2**) views. Abbreviations: hy, hypoflexid; m2, second lower molar; pf, posterofossetid; plf, posterolophid

*General description.* PIMUZ A/V 4204 comprises a damaged left dentary that misses the region caudal to the m2 (Fig. 5A1–A2). The incisor (width: 6.42 mm) shows a flat surface and is still orange (Fig. 5A1). The m1 shows a broken occlusal surface, but the m2 (Mesiodistal length: 8.01, Labiolingual width: 6.50 mm) is partially preserved (Fig. 5A1–A3). Its lingual mesiolingual corner is broken. It is tetralophodont, with lophids oriented linguodistally (Fig. 5A3). The hypoflexid penetrates the tooth distolingually and almost reaches the midline of the tooth. Its tip is opposite to the posterofossetid. The lingual flexid are closed, forming fossetids. The first one is labiolingually shorter than the second and third. The m3 (PIMUZ A/V 4204b) is a protohypsodont teeth, tetralophodont, and shows the three lingual flexids open (Fig. 5B1–B2). The posteroflexid is confluent with the hypoflexid, and the posterolophid is isolated from the other lophids.

*Remarks*. The natural distribution of *Myocastor* encompasses Argentina, Uruguay, Bolivia, Paraguay, and Brazil (Woods et al., 1992). Fossil records of *M. coypus* were reported from Brazil, northern Uruguay, Argentina, and Bolivia (Ameghino, 1902; Boule & Trevenin, 1920; Ferrero & Noriega, 2009; Hoffstetter, 1963; Kerber et al., 2014; Werdelin, 1991) (see Fig. 8 in Kerber et al., 2014). During Pleistocene times, the distribution of *M. coypus* was much wider than its current natural distribution, reaching northeast Brazil in areas where today semiarid conditions are predominant (Kerber et al., 2014; Fig. 8). Kerber et al. (2014) considered the Early Pleistocene species from Santa Fé *Myocastor columnaris* erected by Rusconi (1929) as a valid taxon. On the other hand, other Pleistocene species of *Myocastor* (*e.g., M. minor*, *M. priscus*, and *M. perditus*) were considered probable synonyms of *M. coypus* (Kerber et al., 2014). The material reported here is similar to the extant species. However, it is quite fragmented, and for this reason, we do not identify it at specific level.

## Conclusions

In this work, fossils of three taxa of caviomorph rodents were reviewed. These materials were collected by Santiago Roth in the late nineteenth century in Pleistocene deposits in the Pampas region of Buenos Aires and Santa Fé provinces. The specimens are assigned to *Lagostomus maximus* (Chinchilloidea: Chinchillidae), *Dolichotis* sp. (Cavioidea: Caviidae), and *Myocastor* sp. (Octodontoidea: Echimyidae). Other materials (*Ctenomys* sp.—PIMUZ A/V 4243, Catalog No. 5, specimen 263; *Cavia* sp.—PIMUZ A/V 4218, Catalog No. 5, specimen 263) are not reported here as they probably correspond to sub-recent material.

The fauna of Pleistocene caviomorphs from the Pampean region of Argentina is characterized by the presence of taxa adapted to open environments. This fauna, in addition to the taxa mentioned here, includes the caviids *Cavia*, *Galea*, *Microcavia* and *Neochoerus*, and the ctenomyid *Ctenomys* (Vucetich & Verzi, 1999). Unfortunately, the stratigraphic information of the specimens here reviewed lacks further data, limiting paleoenvironmental and paleobiogeographical considerations. Nevertheless, these fossils emerge as a source of information about the morphology of these taxa during the Pleistocene. Additionally, due to its historical context, the revisiting of the material contributes to the history of one of the most important paleontologists who worked in South America during the second half of the nineteenth century.

## Data Availability

All data generated or analyzed during this study are included in this published article [and its supplementary information files]. 3D models of the specimens PIMUZ/AV 4131 and PIMUZ A/V 4147 are available as supplemental material. The models (.ply) were generated with a 3D Scanner Artec Spider.

## References

[CR1] Ameghino F (1889). Contribución al conocimiento de los mamíferos fósiles de la República Argentina. Actas De La Academia Nacional De Ciencias De La República Argentina.

[CR2] Ameghino F (1902). Notas sobre algunos mamíferos fósiles nuevos o poco conocidas del Valle del Tarija. Anales Del Museo Nacional De Buenos Aires.

[CR3] Antoine P-O, Marivaux L, Croft DA, Billet G, Ganerød M, Jaramillo C, Martin T, Orliac MJ, Tejada J, Altamirano AJ, Duranthon F, Fanjat G, Rousse S, Gismondi RS (2012). Middle Eocene rodents from Peruvian Amazonia reveal the pattern and timing of caviomorph origin and biogeography. Proceedings of the Royal Society B: Biological Sciences.

[CR4] Arnal M, Kramarz AG, Vucetich MG, Frailey CD, Campbell KE (2019). New Paleogene caviomorphs (Rodentia, Hystricognathi) from Santa Rosa, Peru: systematics, biochronology, biogeography and early evolutionary trends. Papers in Palaeontology.

[CR5] Arnal M, Pérez ME, Medina LMT, Campbell KE (2022). The high taxonomic diversity of the Palaeogene hystricognath rodents (Caviomorpha) from Santa Rosa (Peru, South America) framed within a new geochronological context. Historical Biology.

[CR6] Arnaudo ME, Arnal M, Ekdale EG (2020). The auditory region of a caviomorph rodent (*Hystricognathi*) from the early Miocene of Patagonia (South America) and evolutionary considerations. Journal of Vertebrate Paleontology.

[CR7] Bertrand OC, Flynn JJ, Croft DA, Wyss AR (2012). Two new taxa (*Caviomorpha*, *Rodentia*) from the early Oligocene Tinguiririca fauna (Chile). American Museum Novitates.

[CR8] Boivin M, Marivaux L, Antoine P-O (2019). L’apport du registre paléogène d’Amazonie sur la diversification initiale des Caviomorpha (Hystricognathi, Rodentia): implications phylogénétiques, macroévolutives et paléobiogéographiques. Geodiversitas.

[CR9] Boivin M, Marivaux L, Candela AM, Orliac MJ, Pujos F, Salas-Gismondi R, Tejada-Lara J, Antoine P-O (2016). Late Oligocene caviomorph rodents from Contamana. Peruvian Amazonia. Papers in Palaeontology.

[CR10] Boivin M, Marivaux L, Orliac MJ, Pujos F, Salas-Gismondi R, Tejada-Lara JV, Antoine P-O (2017). Late middle Eocene caviomorph rodents from Contamana. Peruvian Amazonia. Palaeontologia Electronica.

[CR11] Boivin M, Marivaux L, Pujos F, Salas-Gismondi R, Tejada-Lara JV, Varas-Malca RM, Antoine P-O (2018). Early Oligocene caviomorph rodents from Shapaja, Peruvian Amazonia. Palaeontographica, Abt A Palaeozoology Stratigraphy..

[CR12] Boule M, Trevenin A (1920). Mammifères fosiles de Tarija Mission Scientifiqué G de Créqui-Montfort et E. Sénéchal de la Grange.

[CR13] Cabrera A (1961). Catálogo de los mamíferos sudamericanos II. Revista Del Museo Argentino De Ciencias Naturales Bernardino Rivadavia, Ciencias Zoológicas.

[CR14] Campbell KE, O’Sullivan PB, Fleagle JG, De Vries D, Seiffert ER (2021). An early Oligocene age for the oldest known monkeys and rodents of South America. Proceedings of the National Academy of Sciences.

[CR15] Campos CM, Tognelli MF, Ojeda RA (2001). *Dolichotis Patagonum*. Mammalian Species.

[CR16] Candela AM, Bonini RA (2017). A new guinea pig (Rodentia, Caviomorpha) from northwestern Argentina: implications for the origin of the genus *Cavia*. Journal of Vertebrate Paleontology.

[CR17] Candela A, Picasso MBJ (2008). Functional anatomy of the limbs of Erethizontidae (Rodentia, Caviomorpha): indicators of locomotor behavior in Miocene porcupines. Journal of Morphology.

[CR18] Cione AL, Tonni EP (1999). Biostratigraphy and chronological scale of upper-most Cenozoic in the Pampean Area, Argentina. Quaternary of South America and Antarctic Peninsula.

[CR19] Cruz LE, Fernicola JC, Carignano C, Bargo MS (2009). Nueva associación faunística del Pleistoceno del este de la Província de Córdoba. Ameghiniana, Suplemento.

[CR20] D’Elía G, Fabre P-H, Lessa EP (2019). Rodent systematics in an age of discovery: recent advances and prospects. Journal of Mammalogy.

[CR21] Dunnum JL, Patton JL, Pardiñas UFJ, Elía GD (2015). Family Caviidae. Mammals of South America Rodents.

[CR22] Eduardo AA, Martinez PA, Gouveia SF, Santos FDS, Aragão WSD, Morales-Barbero J, Kerber L, Liparini A (2018). Extending the paleontology-biogeography reciprocity with SDMs: exploring models and data in reducing fossil taxonomic uncertainty. PLoS ONE.

[CR23] Eisenberg JF, Redford KH (1989). Mammals of the Neotropics—The Central Neotropics.

[CR24] Fabre P-H, Hautier L, Douzery EJP, Hautier L, Cox PG (2015). A synopsis of rodent molecular phylogenetics, systematics and biogeography. Evolution of the Rodents: Advances in Phylogeny, Functional Morphology and Development.

[CR25] Ferreira JD, Negri FR, Sánchez-Villagra MR, Kerber L (2020). Small within the largest: brain size and anatomy of the extinct *Neoepiblema acreensis*, a giant rodent from the Neotropics. Biology Letters.

[CR26] Ferrero BS, Noriega JI, Ribeiro AM, Bauermann CS (2009). La paleontologia de vertebrados en el Quaternario de la Província de Entre Rios (Argentina) estado actual y perspectivas. Quaternário do Rio Grande do Sul e Integrando Conhecimentos.

[CR27] Frailey CD, Campbell KE, Campbell KE (2004). Paleogene rodents from Amazonian Peru: the Santa Rosa Local Fauna. The Paleogene Mammalian fauna of Santa Rosa Amazonian Peru.

[CR28] Giacchino A, Gurovich Y (2001). Homenaje al doctor Santiago Roth a 150 años de su natalicio. Homage to doctor Santiago Roth, 150 years after his birth. Agora Philosophica Revista Marplatense De Filosofía..

[CR29] Gomes ACF, Cartelle C, Lessa G, Kerber L (2019). New fossil remains of Quaternary capybaras (*Rodentia*: *Caviomorpha*: *Caviidae*) from the intertropical region of Brazil: morphology and taxonomy. Journal of South American Earth Sciences.

[CR30] Gómez G, Prado JL, Albedi MT (1999). Micromamiferos del Sitio Arroyo Seco 2 (Provincia de Buenos Aires, Argentina). Sus implicaciones tafonómicas y paleoambientales. Estudios Geológicos.

[CR31] Hoffstetter R (1963). La faune Pléistocène de Tarija (Bolivie). Bulletin Du Muséum National D’histoire Naturell.

[CR32] Jackson JE, Branch LC, Vilarreal D (1996). *Lagostomus Maximus*. Mammal Species.

[CR33] Kerber L (2017). Imigrantes em um continente perdido: O registro fossilífero de roedores Caviomorpha (*Mammalia*: *Rodentia*: *Ctenohystrica*) do Cenozoico do Brasil. Revista Terrae Didatica.

[CR34] Kerber L, Ferreira JD, Negri FR (2019). A reassessment of the cranial morphology of *Neoepiblema acreensis* (Rodentia: Chinchilloidea), a Miocene rodent from South America. Journal of Morphology.

[CR35] Kerber L, Lopes RP, Vucetich MG, Ribeiro AM, Pereira JC (2011). Chinchillidae and Dolichotinae rodents (*Rodentia*, *Hystricognathi*, *Caviomorpha*) from the late Pleistocene of southern Brazil. Revista Brasileira De Paleontologia.

[CR36] Kerber L, Mayer EL, Gomes ACF, Nasif N (2020). On the morphological, taxonomic, and phylogenetic status of South American Quaternary dinomyid rodents (*Rodentia*: *Dinomyidae*). Palaeontologische Zeitschrift.

[CR37] Kerber L, Mayer EL, Ribeiro AM, Vucetich MG (2016). Late Quaternary caviomorph rodents (Rodentia: Hystricognathi) from the Serra da Capivara, northeastern Brazil, with description of a new taxon. Historical Biology.

[CR38] Kerber L, Negri FR, Sanfelice D (2019). Morphology of cheek teeth and dental replacement in the extinct rodent *Neoepiblema* Ameghino, 1889 (Caviomorpha, Chinchilloidea, Neoepiblemidae). Journal of Vertebrate Paleontology.

[CR39] Kerber L, Ribeiro AM, Lessa G, Cartelle C (2014). Late Quaternary fossil record of *Myocastor* Kerr, 1792 (*Rodentia*: *Hystricognathi*: *Caviomorpha*) from Brazil with taxonomical and environmental remarks. Quaternary International.

[CR40] Kerber L, Ribeiro AM, Oliveira EV (2011). The first record of *Galea* Meyen, 1832 (Rodentia, Hystricognathi, Caviidae) in the late Pleistocene of southern Brazil and its paleobiogeographic implications. Alcheringa.

[CR41] Llanos AC, Crespo JA (1952). Ecología de la vizcacha (“Lagostomus maximus maximus” Blaiv.) en el nordeste de la provincia de Entre Ríos. Revista De Investigaciones Agrícolas.

[CR42] Machon, F. (1925). Le géologue Prof. Dr. Santiago Roth, 1850–1924. Verhandlungen der Schweirzerischen Naturforschenden Gesellschaft II, (Teil, Anhahng), 35–41.

[CR43] Madozzo-Jaén MC (2019). Systematic and phylogeny of *Prodolichotis prisca* (Caviidae, Dolichotinae) from the northwest of Argentina (Late Miocene-Early Pliocene): Advances in the knowledge of the evolutionary history of maras. Comptes Rendus Palevol.

[CR44] Madozzo-Jaén MC, Pérez ME, Deschamps DC (2021). The oldest species of *Dolichotis* (Rodentia, Hystricognathi) from the Pliocene of Argentina: redescription and taxonomic status of “*Orthomyctera*” *chapalmalense*. Journal of Mammalian Evolution.

[CR45] Mayer EL, Hubbe A, Kerber L, Haddad-Martim P, Neves W (2016). Taxonomic, biogeographic, and taphonomic reassessment of a large extinct species of paca from the Pleistocene of Brazilfig. Acta Palaeontologica Polonica.

[CR46] Mones A (1982). An equivocal nomenclature: what means hypsodonty?. Paläontologische Zeitschrift.

[CR47] Moore WJ (1981). The mammalian skull.

[CR48] Nasif N, Abdala F (2015). Craniodental ontogeny of the pacarana *Dinomys branickii* Peters 1873 (Rodentia, Hystricognathi, Caviomorpha, Dinomyidae). Journal of Mammalogy.

[CR49] NAV (Nomina Anatomica Veterinaria). (2017). International Committee on Veterinary Gross Anatomical Nomenclature, 6th edition. Committee on Veterinary Gross Anatomical Nomenclature, Editorial Committee Hanover

[CR50] Novacek MJ, Luckett WP, Hartenberger JL (1985). Cranial evidence for rodent affinities. Evolutionary relationships among rodents: a multidisciplinary analysis.

[CR51] Patton JL, Pardiñas UF, D’Elía G (2015). Mammals of South America vol 2 Rodents.

[CR52] Pérez ME (2010). A new rodent (Cavioidea, Hystricognathi) from the middle Miocene of Patagonia, mandibular homologies, and the origin of the crown group Cavioidea *sensu stricto*. Journal of Vertebrate Paleontology.

[CR53] Prado JL, Menegaz AZ, Tonni EP, Salemme MC (1987). Los mamíferos de la Fauna local Paso Otero (Pleistoceno Tardío), Provincia de Buenos Aires. Aspectos Paleoambientales y Bioestratigráficos. Ameghiniana.

[CR54] Quintana CA (1998). Relaciones filogenéticas de roedores Caviinae (Caviomorpha, Caviidae) de América del Sur. Boletín Real De La Sociedad Española De Historia Natural, Sección De Biología.

[CR55] Rasia LL (2021). Reassessing the fossil record of *Lagostomus incisus* Ameghino, 1888 (Rodentia, Caviomorpha) from the late Neogene of southern South America. Publicación Electrónica De La Asociación Paleontológica Argentina.

[CR56] Rasia LL, Bonini RA, Candela AM (2020). Nuevos registros de *Lagostomus* Brookes (Rodentia, Chinchillidae) en el Mioceno Tardío de Argentina y su importancia bioestratigráfica. Andean Geology.

[CR57] Rasia LL, Candela AM (2013). Systematic and biostratigraphic significance of a chinchillid rodent from the Pliocene of eastern Argentina. Acta Palaeontologica Polonica.

[CR58] Rasia LL, Candela AM (2017). Lagostomus telenkechanum sp. nov, a new lagostomine rodent (Caviomorpha, Chinchillidae) from the Arroyo Chasicó Formation (Late Miocene; Buenos Aires Province, Argentina). Journal of Vertebrate Paleontology..

[CR59] Rasia LL, Candela AM (2017). Systematic revision of the vizcachas (Rodentia, Caviomorpha, Chinchillidae) from the Chapadmalal Formation, late Pliocene of Buenos Aires Province, Argentina. Ameghiniana.

[CR60] Rinderknecht A, Blanco RE (2008). The largest fossil rodent. Proceeding of the Royal Society B.

[CR61] Rodrigues PH, Ferigolo J (2004). Roedores pleistocênicos da Planície Costeira do Estado do Rio Grande do Sul. Brasil. Revista Brasileira De Paleontologia.

[CR62] Roth, S. (1889). *Fossiles de la Pampa, Amérique du Sud (Catalogue No.5).* Zürich: Imprimerie Jean Meyer.

[CR63] Rusconi C (1929). Revisión de las especies fósiles argentinas del género Myocastor con descripción de nuevas especies. Anales De La Sociedad Argentina De Estudios Geograficos.

[CR64] Sánchez-Villagra MR, Aguilera O, Horovitz I (2003). The anatomy of the world’s largest extinct rodent. Science.

[CR94] Sánchez-Villagra, M. R., Bond, M., Reguero, M., & Bartoletti, T. J. (2023). From fossil trader to palaeontologist: On Swiss-born naturalist Santiago Roth and his scientific contributions. *Swiss Journal of Palaeontology,**this volume*. 10.1186/s13358-023-00282-6 PMC10495517 37706073

[CR65] Sarrat CM (2009). Nueva localidad fosilífera del Pleistoceno de la provincia de Córdoba, Argentina: implicancias bioestratigráficas. Ameghiniana, Suplemento.

[CR66] Scillato-Yané, G.J., Tonni, E.P., Carlini, A.A., & Noriega, J.I. (1998). Nuevos hallazgos de mamíferos del Cuaternario en el arroyo Toropí, Corrientes, Argentina. Aspectos bioestratigráficos, paleoambientaisy paleozoogeográficos. *In* Congreso Latinoamericano de Geología, 10/Congreso Nacional De Geología Económica, 6. Buenos Aires, Actas, Buenos Aires.

[CR67] Spotorno AE, Patton JL, Patton JL, Pardinas UFJ, D'Elia G (2015). Superfamily Chinchilloidea Bennett, 1833. Mammals of South America: Rodents.

[CR68] Tonni EP (1981). *Pediolagus salinicola* (Rodentia, Caviidae) en el Pleistoceno Tardío de la Provincia de Buenos Aires. Ameghiniana.

[CR69] Tonni EP, Bargo MS, Prado JL (1988). Los cambios ambientales en el Pleistoceno Tardío y Holoceno del sudeste de la Provincia de Buenos Aires a través de una secuencia de mamíferos. Ameghiniana.

[CR70] Tonni EP, Fidalgo F (1982). Geología y Paleontología de los sedimentos del Pleistoceno en el area de Punta Hermengo (Miramar, Prov. de Buenos Aires, Rep. Argentina): aspectos paleoclimáticos. Ameghiniana.

[CR71] Ubilla M, Oliveira EV, Rinderknecht A, Pereira J (2008). The hystricognath rodent Microcavia in the Late Pleistocene of Brazil (Rio Grande do Sul, South America) (Mammalia: *Caviidae*). Biogeographic and paleoenviromental implications. Neues Jahrbuch Für Geologie Und Paläontologie Abhandlungen..

[CR72] Ubilla M, Perea D, Aguilar CG, Lorenzo N (2004). Late Pleistocene vertebrates from northern Uruguay: tools for biostratigraphic, climatic and environmental reconstruction. Quaternary International.

[CR73] Ubilla M, Perea D, Rinderknecht A, Corona A, Ribeiro AM, Bauermann SG, Scherer CS (2009). Pleistocene mammals from Uruguay: biostratigraphic, biogeographic and environmental connotations. Quaternário do Rio Grande do Sul-Integrando Conhecimentos.

[CR74] Ubilla M, Rinderknecht A (2001). Consideraciones sobre el género *Galea* Meyen, 1831 (*Rodentia*, *Caviidae*), su registro en el Pleistoceno de Uruguay y descripción de una nueva especie extinguida. Boletín De La Sociedad Española De Historia Natural.

[CR75] Ubilla M, Rinderknecht A (2003). A Late Miocene Dolichotinae (Mammalia, Rodentia, Caviidae) from Uruguay, with comments about the relationships of some related fossil species. Mastozoología Neotropical.

[CR76] Ubilla M, Rinderknecht A (2014). Comparative analysis of *Galea* (*Rodentia, Caviidae*) and expanded diagnosis of *Galea ortodonta* Ubilla and Rinderknecht, 2001 (Late Pleistocene, Uruguay). Geobios.

[CR77] Ubilla M, Rinderknecht A (2016). *Lagostomus maximus* (Desmarest) (Rodentia, Chinchillidae), the extant plains vizcacha in the Late Pleistocene of Uruguay. Alcheringa.

[CR78] Upham NS, Patterson BD, Vassallo AI, Antenucci D (2015). Evolution of the caviomorph rodents: a complete phylogeny and time tree of living genera. Biology of caviomorph rodents: diversity and evolution.

[CR79] Vezzosi R, Kerber L (2017). The southernmost record of a large erethizontid rodent (Hystricomorpha: Erethizontoidea) in the Pleistocene of South America: biogeographic and paleoenvironmental implications. Journal of South American Earth Sciences.

[CR80] Voglino, D. Carrillo-Briceño J. D., Furrer H., Balcarcel A., Rangel De Lazaro G., Aguirre Fernandez G., & Forasiepi A. M. (2023). Pampean megamammals in Europe: The fossil collections from Santiago Roth. *Swiss Journal of Palaeontology,**this volume*. 10.1186/s13358-023-00283-5 PMC10542304 37790996

[CR81] Vucetich MG, Arnal M, Deschamps CM, Pérez ME, Vieytes EC, Vassallo A, Antonucci D (2015). A brief history of caviomorph rodents as told by the fossil record. Biology of caviomorph rodents: diversity and evolution.

[CR82] Vucetich MG, Verzi DH (1999). Changes in diversity and distribution of the caviomorph rodents during the Late Cenozoic in South America. Quaternary of South America and Antarctic Peninsula.

[CR83] Vucetich MG, Verzi DH (2002). First record of Dasyproctidae (Rodentia) in the Pleistocene of Argentina. Paleoclimatic implication. Palaeogeography, Palaeoclimatology, Palaeoecology.

[CR84] Vucetich MG, Verzi DH, Hartenberger JL (1999). Review and analysis of the radiation of the South American Hystricognathi (Mammalia, Rodentia). Comptes Rendus De L'académie Des Sciences Series IIA Earth and Planetary Science.

[CR85] Vucetich MG, Verzi DH, Tonni EP (1997). Paleoclimatic implications of the presence of *Clyomys* (Rodentia, Caviomorpha) in the Upper Pliocene of Buenos Aires Province. Palaeogeography, Palaeoclimatology, Palaeoecology.

[CR86] Wahlert JH, Luckett WP, Hartenberger J-L (1985). Cranial foramina of rodents. Evolutionary relationships among rodents: A multidisciplinary analysis.

[CR87] Weigerlt G (1951). Santiago Roth 1850–1924, Ein Berner als Wissenschaftlicher pionier in Südamerika. Berner Zeitschrift Für Geschichte Und Heimatkunde.

[CR88] Werdelin, L. (1991). Pleistocene vertebrates from Tarija, Bolivia in the collections of the Swedish Museum of Natural History. In R. Suarez-Soruco (Eds.), Fosiles y Facies de Bolivia, Vol. I Vertebrados (pp. 673–684). Santa Cruz: Revista Técnica de YPFB 12 (3–4).

[CR89] Wible JR (2010). Petrosal anatomy of the nine-banded armadillo, *Dasypus novemcinctus* Linnaeus, 1758 (Mammalia, Xenarthra, Dasypodidae). Annals of Carnegie Museum.

[CR90] Wible JR, Shelley S (2020). Anatomy of the petrosal and middle ear of the brown rat, *Rattus norvegicus* (Berjenhout, 1769) (Rodentia, Muridae). Annals of Carnegie Museum.

[CR91] Wible JR, Wang Y, Dawson MR (2005). Cranial anatomy and relationships of a new ctenodactyloid (Mammalia, Rodentia) from the early Eocene of Hubei Province, China. Annals of Carnegie Museum.

[CR92] Wilson L, Sánchez-Villagra M (2009). Heterochrony and patterns of cranial suture closure in hystricognath rodents. Journal of Anatomy.

[CR93] Woods CA, Contreras L, Willner-Chapman G, Whidden HP (1992). *Myocastor Coypus*. Mammalian Species.

